# The Role of Plasma Neurofilament Light Protein for Assessing Cognitive Impairment in Patients With End-Stage Renal Disease

**DOI:** 10.3389/fnagi.2021.657794

**Published:** 2021-05-28

**Authors:** Yi-Chou Hou, Chuen-Lin Huang, Chien-Lin Lu, Cai-Mei Zheng, Yuh-Feng Lin, Kuo-Cheng Lu, Ya-Lin Chung, Ruei-Ming Chen

**Affiliations:** ^1^Graduate Institute of Clinical Medicine, College of Medicine, Taipei Medical University, Taipei, Taiwan; ^2^Department of Internal Medicine, Cardinal Tien Hospital, New Taipei City, Taiwan; ^3^School of Medicine, Fu Jen Catholic University, New Taipei City, Taiwan; ^4^Department of Medical Research, Cardinal Tien Hospital, New Taipei City, Taiwan; ^5^Department of Physiology and Biophysics, National Defense Medical Center, Graduate Institute of Physiology, Taipei, Taiwan; ^6^Department of Nephrology, Fu Jen Catholic University Hospital, New Taipei City, Taiwan; ^7^Division of Nephrology, Department of Internal Medicine, Taipei Medical University Shuang Ho Hospital, New Taipei City, Taiwan; ^8^Division of Nephrology, Department of Internal Medicine, School of Medicine, College of Medicine, Taipei Medical University, Taipei, Taiwan; ^9^Taipei Medical University-Research Center of Urology and Kidney, Taipei Medical University, Taipei, Taiwan; ^10^National Defense Medical Center, Graduate Institute of Medical Sciences, Taipei, Taiwan; ^11^Department of Nephrology, Taipei Tzu Chi Hospital, Buddhist Tzu Chi Medical Foundation, New Taipei City, Taiwan; ^12^Department of Medical Laboratory, Cardinal-Tien Hospital, New Taipei City, Taiwan; ^13^TMU Research Center of Cancer Translational Medicine, Graduate Institute of Medical Sciences, College of Medicine, Taipei Medical University, Taipei, Taiwan; ^14^Cell Physiology and Molecular Image Research Center, Wan Fang Hospital, Taipei Medical University, Taipei, Taiwan; ^15^Anesthesiology and Health Policy Research Center, Taipei Medical University Hospital, Taipei, Taiwan

**Keywords:** cognitive impairment, dementia, ESRD, neurofilament light chain, amyloid beta, tau

## Abstract

**Introduction:** End-stage renal disease (ESRD) is defined as the irreversible loss of renal function, necessitating renal replacement therapy. Patients with ESRD tend to have more risk factors for cognitive impairment than the general population, including hypertension, accumulative uremic toxin, anemia, and old age. The association between these risk factors and the pathologic protein was lacking. Blood-based assays for detecting pathologic protein, such as amyloid beta (Aβ), total tau protein, and neurofilament light chain (NfL), have the advantages of being less invasive and more cost-effective for diagnosing patients with cognitive impairment. The aim of the study is to validate if the common neurologic biomarkers were different in ESRD patients and to differentiate if the specific biomarkers could correlate with specific correctable risk factors.

**Methods:** In total, 67 participants aged >45 years were enrolled. The definition of ESRD was receiving maintenance hemodialysis for >3 months. Cognitive impairment was defined as a Mini-Mental State Examination score of <24. The participants were divided into groups for ESRD with and without cognitive impairment. The blood-based biomarkers (tau protein, Aβ1/40, Aβ1/42, and NfL) were analyzed through immunomagnetic reduction assay. Other biochemical and hematologic data were obtained simultaneously.

**Summary of results:** The study enrolled 43 patients with ESRD who did not have cognitive impairment and 24 patients with ESRD who had cognitive impairment [Mini-Mental State Examination (MMSE): 27.60 ± 1.80 vs. 16.84 ± 6.40, *p* < 0.05]. Among the blood-based biomarkers, NfL was marginally higher in the ESRD with cognitive impairment group than in the ESRD without cognitive impairment group (10.41 ± 3.26 vs. 8.74 ± 2.81 pg/mL, *p* = 0.037). The concentrations of tau protein, amyloid β 1/42, and amyloid β 1/40 (*p* = 0.504, 0.393, and 0.952, respectively) were similar between the two groups. The area under the curve of NfL to distinguish cognitively impaired and unimpaired ESRD patients was 0.687 (95% confidence interval: 0.548–0.825, *p* = 0.034). There was no correlation between the concentration of NfL and MMSE among total population (*r* = −0.153, *p* = 0.277), patients with (*r* = 0.137, *p* = 0.583) or without cognitive impairment (*r* = 0.155, *p* = 0.333).

**Conclusion:** Patients with ESRD who had cognitive impairment had marginally higher plasma NfL concentrations. NfL concentration was not correlated with the biochemical parameters, total MMSE among total population or individual groups with or without cognitive impairment. The concentrations of Aβ1/40, Aβ1/42, and tau were similar between the groups.

## Introduction

Chronic kidney disease (CKD) is defined as the progressive loss of glomerular infiltration. CKD etiologies include diabetes mellitus, hypertension, dyslipidemia, hereditary kidney diseases such as polycystic kidney disease, and chronic exposure to nephrotoxic agents such as non-steroidal anti-inflammatory drugs. The progressive loss of glomerular filtration rate (GFR) induces multiple comorbidities, such as fluid retention, electrolyte imbalance, vitamin D deficiency, renal anemia mediated through insufficient erythropoietin (EPO) production by the kidney, and uremic toxin accumulation (Inker et al., 2014). The aforementioned comorbidities dysregulate the homeostasis of various organs and contribute to multiple disorders, such as vascular calcification, left ventricular hypertrophy, renal osteodystrophy, immune dysfunction, sarcopenia, and ischemic stroke (Li et al., 2015; Hou et al., 2017; Cherng et al., 2018). Renal replacement therapy, including hemodialysis, peritoneal dialysis, and kidney transplantation, was developed to remove excessive body fluid and uremic toxins to restore the homeostasis of the body in patients with end-stage renal disease (ESRD) (Mendu and Weiner, 2020). However, renal placement therapy also leads to several complications, such as immune dysregulation, malnutrition, arterial stiffness (Maraj et al., 2018), and insufficient toxin clearance because of the modality of renal replacement therapy and residual renal function preservation (van Gelder et al., 2020).

Clinically, cognitive impairment is recognized as progressive decline of cognitive, behavioral and sensorimotor function, which would therefore impair the memory and activity of daily life (Geda, 2012). Memory loss influences independent daily activities and further causes psychological stress and depressive mood. The screening for cognitive impairment is mostly based on psychiatric scales such as the Mini-Mental State Examination (MMSE), clinical dementia rating, and Montreal Cognitive Assessment (MoCA) (Malek-Ahmadi et al., 2014). The etiologies of cognitive impairment or dementia can be divided into vascular cognitive impairment, neurodegenerative disorders associated with the prion-like spreading, deposition of misfolded proteins (such as amyloid-beta, alfa-synuclein, hyperphosphorylated tau, transactive response DNA-binding protein 43 (TDP-43) or Lewy body) or frontotemporal dementia (Soto and Satani, 2011; Twohig and Nielsen, 2019; D’errico and Meyer-Luehmann, 2020; Hall et al., 2020; Jo et al., 2020). Cognitive impairment/dementia is commonly observed in patients with CKD. The incidence of cognitive impairment among patients with ESRD was reported to be 10–40%, which is higher than that among the general population (Drew et al., 2019). A study based on the National Health Insurance research database in Taiwan showed that the incidence of dementia was higher among patients with ESRD among in those without ESRD (Kuo et al., 2019). Patients with CKD tend to have more risk factors for dementia than members of the general population, including hypertension, diabetes mellitus, old age, and dyslipidemia (Chen et al., 2017b). In patients with CKD, the brain parenchyma may be injured, which induces vascular cognitive impairment because of insufficient blood flow due to ischemia, anemia associated with insufficient EPO (Hung et al., 2019), and intradialytic cerebral ischemia (MacEwen et al., 2017). Additionally, brain parenchymal cells might be damaged directly by uremic toxins, which could diffuse across the blood–brain barrier (e.g., indoxyl sulfate; Lin et al., 2019). Cohort studies have shown that plasma Aβ is associated with Alzheimer’s disease (Lue et al., 2017). From the cohort studies for CKD patients, the plasma concentration of pathologic protein for cognitive impairment, such as Aβ, was negatively correlated with the glomerular filtration rates (Gronewold et al., 2016), and the Aβ concentration was higher in the CKD patients with cognitive impairment than CKD patients without cognitive impairment (Vinothkumar et al., 2017). In patients with CKD, cognitive impairment is prevalent and cognitive impairment severity influences clinical outcomes; therefore, identifying a biomarker for the early detection of cognitive disorders and prediction of cognitive impairment severity is important.

For the early detection of cognitive impairment, biomarkers have been developed in different fields, including neuropsychological tests, neuroimaging, or biomarker detection in cerebrospinal fluid (CSF). Amyloid-beta peptides such as Aβ42 and Aβ40, and phosphorylated tau in CSF are well-validated biomarkers for the diagnosis of AD in clinical routine. In this regard several studies showed good correlations between the CSF biomarker levels and the correspondent neuropathological changes in AD brains (Tapiola et al., 2009; Lue et al., 2017; Baiardi et al., 2019). Neurofilament light chain (NfL), which is the main component of the cytoskeleton of myelinated neuron axons, is released from the damaged axons. NfL expression reflects subcortical neuronal damage and white matter damage (Gaetani et al., 2019). NfL in CSF increases in neurodegenerative diseases, such as Alzheimer disease, frontotemporal dementia, vascular dementia and even human-immunodeficiency virus associated cognitive impairment (Abu-Rumeileh et al., 2018; Anderson et al., 2018; Chatterjee et al., 2019; Martin et al., 2019). Akamine et al. (2020) demonstrated that the concentration of NfL increased along with the serum creatinine. The NfL is sensitive to detect the neuroaxonal damage, but it is not highly specific as overlapping levels exist among different neurodegenerative diseases except amyotrophic lateral sclerosis (Jeppsson et al., 2019; Verde et al., 2019; Gaetani et al., 2019). The report from Mattsson et al. (2017) demonstrated that the concentration of NfL in plasma was associated with severity of cognitive impairment. Plasma NfL was also associated also with other relevant variables such as neuroradiological markers of neurodegeneration, disease severity and survival in other neurodegenerative diseases such as frontotemporal dementia, Parkinson disease and prion disease (Lin et al., 2018; Abu-Rumeileh et al., 2020; Benussi et al., 2020; Spotorno et al., 2020). Moreover, plasma NfL was elevated in animal models of neurodegenerative disorders such as AD or Parkinson’s disease (Loeffler et al., 2020). In the CSF of AD patients, the concentrations of pathologic proteins in CSF were sub-ng/mL or several ng/mL; their measured concentration in the blood might be as low as the pg/mL range (Yang et al., 2017). Therefore, ultrasensitive techniques should be applied, such as immunomagnetic reduction (IMR), for detecting them (Yang et al., 2017). In this cohort, we used IMR instead of enzyme-linked immunoassay (ELISA) or other methods. IMR involves the use of magnetic beads to pull down target molecules for increased sensitivity and specificity.

The aim of the study is to validate if the common neurologic biomarkers, such as Aβ, tau protein, and NfL, could differentiate the cognitive impairment in ESRD patients by immunomagnetic reduction. Besides, we would like to correlate the neurologic biomarkers with other clinical parameters in order to search for correctable risk factors for cognitive impairment in ESRD patients.

## Materials and Methods

### Study Subjects

This study was conducted at a regional hospital in New Taipei City, Taiwan in accordance with the tenets outlined in the Declaration of Helsinki. The study protocol was approved by the Ethics Committee of Human Studies at Cardinal Tien Hospital (CTH-108-2-5-002). The study period was from August 2019 to December 2020. Patients receiving maintenance hemodialysis (three times per week) continuously for > 3 months were enrolled. All patients received conventional hemodialysis using a high-flux or high efficient dialyzer. The exclusion criteria were as follows: (1) age <45 years; (2) stroke within 6 months; and (3) aphasic, illiterate, or unable to write in or understand Chinese or Taiwanese. We obtained written informed consent from enrolled participants. Subsequently, patients were divided into two groups based on their MMSE score. Blood and urine samples were obtained. Demographic data were obtained from medical records in Cardinal Tien Hospital. Diagnoses of congestive heart failure, diabetes mellitus, and hypertension were confirmed using medical records. Stroke diagnosis 6 months before enrollment was confirmed. Body weight and height were measured after hemodialysis and body mass index was obtained. Pre-dialytic hematologic and biochemical parameters were obtained within the month after informed consent was obtained on the mid-day (Wednesday or Thursday) as follows: hemoglobin, platelet count, white blood cell count, glutamic oxaloacetic transaminase (GOT), glutamic pyruvic transaminase (GPT), albumin, blood sugar, uric acid, total cholesterol, triglyceride, sodium, potassium, calcium, phosphorus and intact parathyroid hormone. Estimated glomerular filtration rate (eGFR) was determined by the Modification of Diet in Renal Disease Study equation (Levey et al., 2006). Serum urea levels were recorded pre- and postdialysis to calculate single-pool fractional clearance of urea (Kt/V), which serves as the parameter of adequacy for dialysis (Daugirdas, 1995). The normalized protein catabolic rate (nPCR), as the parameter of dietary protein intake, was calculated by applying the 2-blood urea nitrogen (BUN) method for the predialysis BUN level from monthly kinetic modeling sessions, and the Daugirdas-Schniditz rate equation was used to estimate of the equilibrated postdialysis BUN level (Daugirdas, 1995).

#### Sample Collection

A non-fasting venous blood sample was drawn from each participant and then deposited in a dipotassium ethylenediaminetetraacetic acid (K2 EDTA) tube. The total volume of blood obtained was 10 mL. The sample was drawn before hemodialysis treatment midweek (Wednesday or Thursday). The blood samples were centrifuged at 2,500 × g for 15 min within 3 h of collection, and plasmas were aliquoted into cryotubes (1 mL per tube) and stored at –80°C (Liu et al., 2020). Each sample was given an identification number after collection. NfL, Aβ1/40, Aβ1/42, and tau concentrations were measured using IMR technology for all the collected plasma samples. The reagent for magnetic nanoparticles was dispersed in a phosphoryl buffer solution at pH 7.2.

#### IMR Measurement

These immobilizing antibodies for reagent nanoparticles were produced for NfL (Santa Cruz/sc20011), Aβ1/40 (Sigma/A3981), Aβ1/42 (Abcam/ab34376), and tau protein (Sigma/T9450). The mean diameter of antibody functionalized magnetic nanoparticles was 50–60 nm. The magnetic concentration of each type of reagent was 12 mg-Fe/mL, except for NfL (10 mg/Fe/mL). The compositions of IMR reagents in the human plasma were as follows: 80 μL of Aβ1/40 reagent (MF-AB0–0060, MagQu) mixed with 40 μL of human plasma, 60 μL of Aβ1/42 reagent (MFAB2–0060, MagQu) with 60 μL of human plasma, 80 μL of tau reagent (MF-TAU-0060, MagQu) with 40 μL of human plasma, and 60 μL of NfL reagent with 60 μL of sample (Lue et al., 2017; Liu et al., 2020). The IMR reagent and human plasma were mixed at room temperature. A superconducting quantum interference device based on a magnetic susceptometer (XacPro-S MagQu) was used to detect the reduction in magnetic susceptibility. Biomarker concentrations were transformed from IMR signals. The detection range for total tau protein, Aβ42, Aβ40, and NfL was 0.1∼3,000 pg/mL, 1∼30,000 pg/mL, 1∼1,000 pg/mL, and 0.0033 ∼1,000 pg/mL respectively. The intra-assay or inter-assay coefficient of variation for assaying Aβ1-40, Aβ1-42, total-Tau, or NfL using IMR is within the range of 7–10%. The mean value for each sample was used for statistical analysis.

#### Cognitive Assessment

The Chinese version of the MMSE was employed by the same doctor for the entire study (Li et al., 2016). Cognitive impairment was defined as MMSE scores of 10–24. The normal cognitive function was defined as MMSE scores of 25–30 (Folstein et al., 1975). The MMSE was taken within the first hour of hemodialysis to avoid hemodynamic variation mediated by ultrafiltration.

### Statistics

Continuous variables are presented as means ± standard deviations. Categorical values are expressed as the frequency of count and the percentage. Mann-Whitney Rank Sum Test was used for comparisons of continuous variables between the two groups, including biochemical and laboratory data, plasma neurologic biomarkers and the interaction of NfL with other neurologic biomarkers. The chi-square test was used to analyze the association between the category variables. Spearman’s rank correlation coefficient was used to correlate NfL concentration with biochemical and hematologic results, the total and individual categories of the MMSE and the MMSE of participants with and without cognitive impairment. Receiver operating characteristic (ROC) curve analysis was conducted to differentiate the cognitive impairment in ESRD patients by NfL. Delong’s non-parametric method was used to determine the confidence interval (CI) for the area under the curve (AUC), sensitivity, and specificity. All statistical analyses were performed using the statistical package SPSS for Windows (Version XVII; SPSS, Inc., Chicago, IL, United States). A two-sided *P*-value of < 0.05 was considered statistically significant.

## Results

Table 1 presented the demographic characteristics of the participants. In total, 67 participants were enrolled in the study. Of these, 24 participants had ESRD with cognitive impairment and 43 participants had ESRD without cognitive impairment (control group). Participants in the cognitive impairment group were older than those in the control group (74.80 ± 7.42 vs. 67.34 ± 8.45 years, *p* < 0.05). The proportion of women was higher in the cognitive impairment group than in the control group (60.3% vs. 21.9%, *p* < 0.05). In the patients with cognitive impairment, the percentage of educations year less than 6 years was higher than the patients without cognitive impairment (66.7% vs. 32.5%, p < 0.05). The educations years more than 10 years was higher in the patients without cognitive impairment than patients with cognitive impairment (58.1% vs. 12.5%, *p* < 0.05). Table 2 presents the biochemical and hematological results of the participants. Predialysis creatinine (6.77 ± 1.93 mg/dL vs. 9.13 ± 2.28 mg/dL, *p* < 0.05), albumin (3.68 ± 0.26 g/dL vs. 3.90 ± 0.30 g/dL, *p* < 0.05), phosphorus (4.55 ± 1.10 mg/dL vs. 5.32 ± 1.56 mg/dL, *p* < 0.05) and hemoglobin (9.94 ± 1.34 g/dL vs. 10.16 ± 1.29 g/dL, *p* < 0.05) levels were lower in the cognitive impairment group than in the control group. The estimated GFR was higher in the cognitive impairment group (7.70 ± 3.03 mL/min/1.73 m^2^ vs. 6.36 ± 2.89 mL/min/1.73 m^2^, *p* < 0.05). Furthermore, the potassium concentration, white blood cell count, and platelet count were lower in the cognitive impairment group than in the control group, although the mean values were within the reference range. The Kt/V and normalized protein catabolic rate were similar between the groups.

**TABLE 1:** Demographic characteristics of the participants.

	**ESRD without cognitive impairment**	**ESRD with cognitive impairment**
Sample sizes	43	24
Age (year-old)	67.3 ± 8.5	74.8 ± 7.4*
Education level *		
0–6 years	14 (32.5%)	16 (66.7%)
7–9 years	4 (9.3%)	5 (20.8%)
≥10 years	25 (58.1%)	3 (12.5%)
Gender (female)	9 (22%)	14 (60%)*
Diabetes mellitus	24 (58%)	18 (78%)*
Hypertension	27 (66%)	13 (57%)*
Congestive heart failure	5 (12%)	7 (30%)
Stroke	4 (10%)	3 (13%)
BMI	25.1 ± 4.1	25.4 ± 5.4

ESRD, end-stage renal disease; BMI, body mass index. *p < 0.05.

**TABLE 2:** Biochemical and laboratory data of the participants.

	**ESRD without cognitive impairment**	**ESRD with cognitive impairment**
Sample sizes	43	24
Blood urea nitrogen (mg/dL)	72 ± 18	61 ± 23
Creatinine (mg/dL)	9.13 ± 2.28	6.77 ± 1.93*
eGFR (mL/min/1.73 m^2^)	6.36 ± 2.89	7.70 ± 3.03*
Sodium (mEq/L)	138 ± 3	137 ± 5
Potassium (mEq/L)	4.66 ± 0.70	4.20 ± 0.70*
Albumin (g/dL)	3.90 ± 0.30	3.68 ± 0.26*
Calcium (mg/dL)	8.95 ± 0.69	9.06 ± 0.81
Phosphorus (mg/dL)	5.32 ± 1.56	4.55 ± 1.10*
Blood sugar (mg/dL)	152 ± 65	169 ± 86
Triglyceride (mg/dL)	150 ± 107	144 ± 75
Total cholesterol (mg/dL)	149 ± 28	163 ± 86
Uric acid (mg/dL)	6.43 ± 1.84	6.45 ± 1.56
Intact parathyroid hormone (pg/mL)	357 ± 306	475 ± 400
White blood cell (/μL)	6,591 ± 1,932	5,367 ± 1,144*
Hemoglobin (g/dL)	10.16 ± 1.29	9.94 ± 1.34*
Platelet count (/μL)	182 ± 60	173 ± 44*
Kt/V	1.47 ± 0.80	1.48 ± 0.62
nPCR	1.07 ± 0.31	1.10 ± 0.43

ESRD, end-stage renal disease; eGFR, estimated glomerular filtration; Kt/V, single-pool fractional clearance of urea; nPCR, normalized protein catabolic rate. *p < 0.05.

**FIGURE 1:**
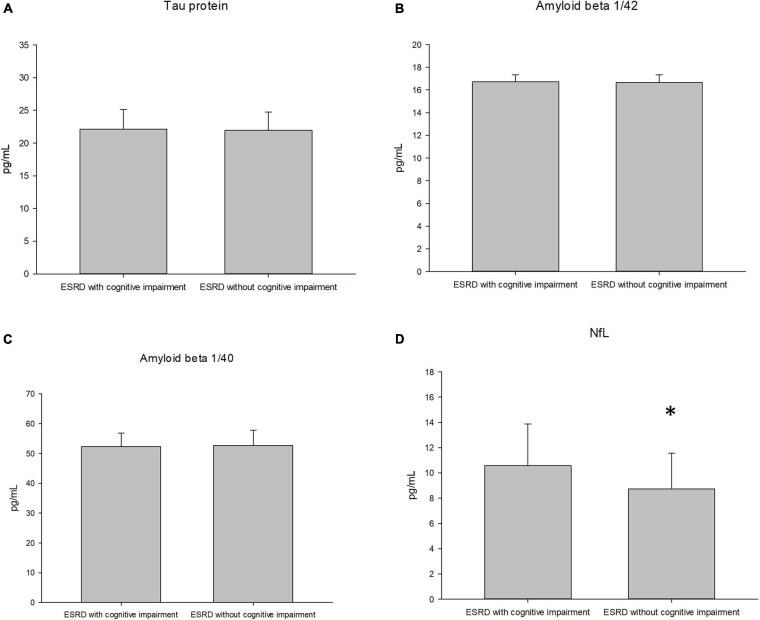
The plasma concentration of the tau protein **(A)**, amyloid 1/42 **(B)**, amyloid 1/40 **(C)**, and neurofilament light chain [NfL, **(D)**] between groups of the subjects.

**FIGURE 2:**
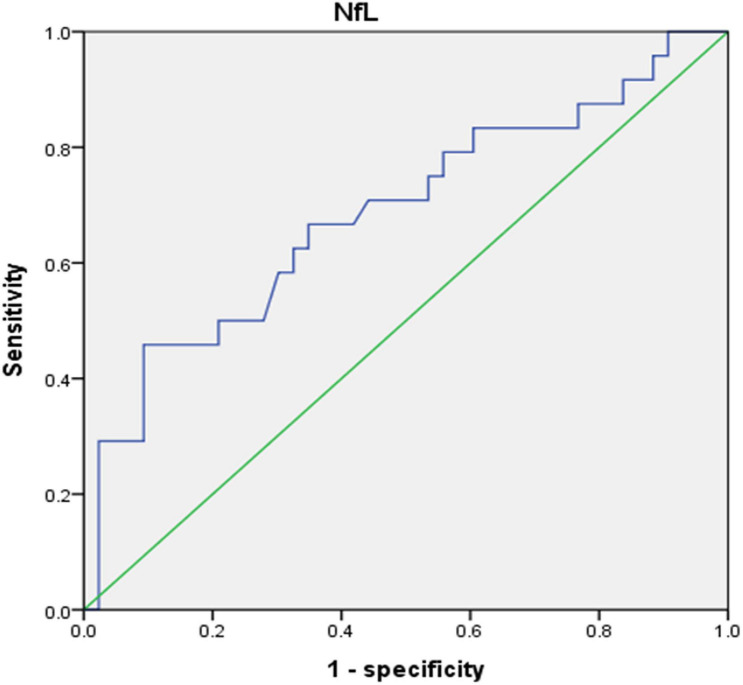
The ROC curve of the NfL for cognitive impairment of in ESRD.

Table 3 presents a comparison of neurological biomarkers between the groups. The NfL concentration was marginally higher in the cognitive impairment group than in the control group (10.65 ± 3.23 pg/mL vs. 8.66 ± 2.76 pg/mL, *p* = 0.03). Furthermore, the concentrations of total tau protein, Aβ1/42 and Aβ1/40 didn’t differ between groups (*p* = 0.45, 0.35 and 0.91, respectively). Figure 1 displays a comparison of neurological biomarkers between the groups. Figure 2 displays the receiver operating characteristic curve of NfL to differentiate the cognitively impaired and unimpaired ESRD patients. The AUC for NfL was 0.687 (*p* = 0.012, 95% CI: 0.548–0.825).

**TABLE 3:** Plasma neurological biomarkers of the participants.

	**ESRD without cognitive impairment**	**ESRD with cognitive impairment**
Sample sizes	43	24
Tau protein, pg/mL	21.89 ± 2.77	22.03 ± 2.94
Amyloid beta 1/42, pg/mL	16.63 ± 0.65	16.69 ± 0.61
Amyloid beta 1/40, pg/mL	53.10 ± 5.30	52.50 ± 4.66
NfL, pg/mL	8.66 ± 2.76	10.65 ± 3.23*

ESRD, end-stage renal disease; NfL, neurofilament light chain. *p < 0.05.

Table 4 demonstrated the correlation between NfL and age and hematologic and biochemical data. As the concentration of NfL might correlate with the age (Vågberg et al., 2015), we performed the correlation between NfL and age (*r* = −0.081, *p* = 0.530). There was no correlation between the hematologic and biochemical parameters with NfL.

**TABLE 4:** the correlation between and NfL and the biochemical/hematologic data in total population.

	**Coefficient of correlation**	***p*-value**
Age	–0.081	0.530
Blood urea nitrogen (mg/dL)	0.131	0.373
Creatinine (mg/dL)	0.032	0.800
eGFR (mL/min/1.73 m^2^)	0.057	0.790
Sodium (mEq/L)	–0.032	0.804
Potassium (mEq/L)	0.105	0.411
Albumin (g/dL)	0.088	0.492
Calcium (mg/dL)	0.146	0.279
Phosphorus (mg/dL)	–0.026	0.842
Blood sugar (mg/dL)	–0.084	0.525
Triglyceride (mg/dL)	–0.197	0.158
Total cholesterol (mg/dL)	0.095	0.498
Uric acid (mg/dL)	–0.131	0.338
Intact parathyroid hormone (pg/mL)	0.036	0.390
White blood cell (/μL)	–0.108	0.408
Hemoglobin (g/dL)	0.063	0.632
Platelet count (/μL)	0.00	0.998
		

Table 5 presents a comparison of the two groups for different categories of the MMSE. The scores of orientation to time (2.29 ± 1.04 vs. 4.51 ± 0.66, *p* < 0.05) and place (3.41 ± 1.90 vs. 4.97 ± 0.15, *p* < 0.05), registration, attention and calculation, recall, and language were lower in patients who had ESRD with cognitive impairment than in those who had ESRD without cognitive impairment.

**TABLE 5:** Mini-mental state examination results of the participants.

	**ESRD without cognitive impairment**	**ESRD with cognitive impairment**
Sample sizes	43	24
MMSE (30 points maximum)	27.6 ± 1.8	18.0 ± 5.1*
Orientation to time (5 points maximum)	4.51 ± 0.66	2.29 ± 1.04*
Orientation to place (5 points maximum)	4.97 ± 0.15	3.41 ± 1.90*
Registration (3 points maximum)	2.97 ± 0.15	2.62 ± 0.57*
Attention and calculation (5 points maximum)	4.34 ± 1.08	1.45 ± 1.53*
Recall (3 points maximum)	2.44 ± 0.62	1.29 ± 1.04*
Language (9 points maximum)	8.27 ± 1.00	6.33 ± 1.80*

ESRD, end-stage renal disease; MMSE, Mini-Mental State Examination. *p < 0.05.

**FIGURE 3:**
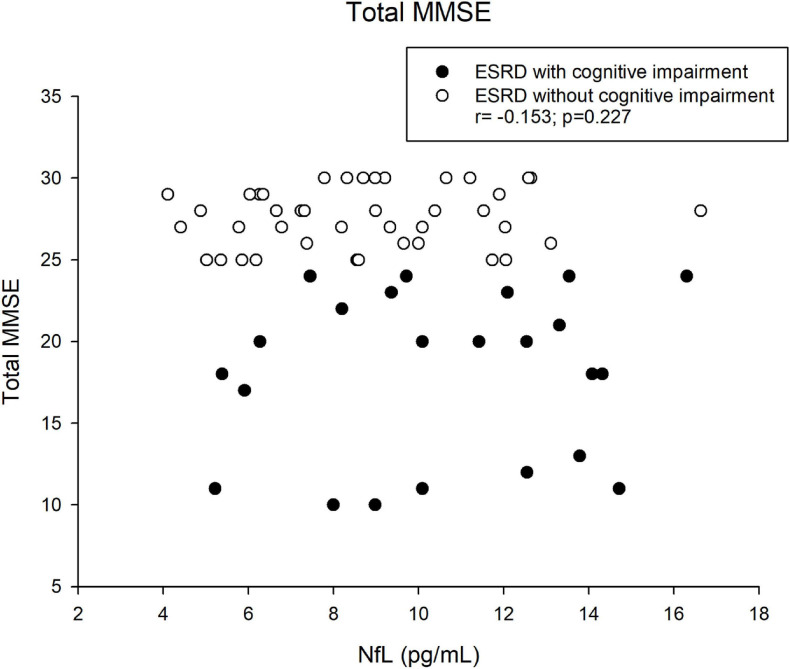
Correlation between NfL and total MMSE in ESRD patients.

**FIGURE 4:**
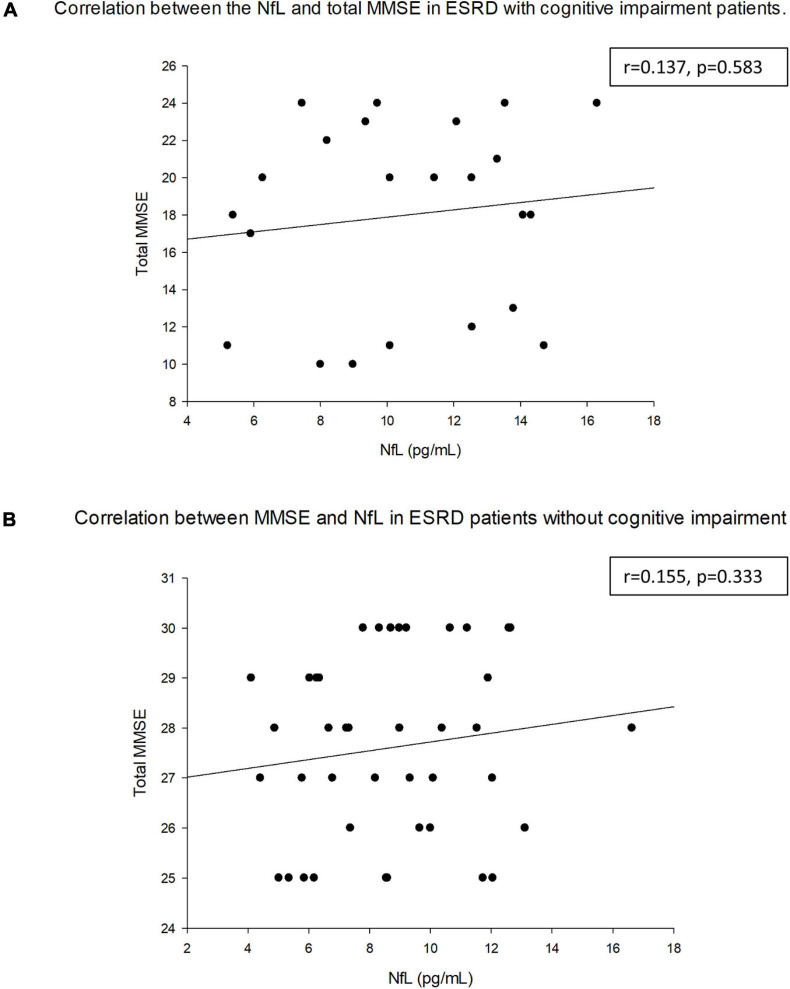
Correlation between NfL and total MMSE with **(A)** and without **(B)** cognitive impairment.

Table 6 demonstrated the correlation between NfL and MMSE (total and individual categories. The correlation coefficient between NfL and the category of orientation to place was –0.262 (*p* = 0.036). No correlation was observed with other subsets. Figure 3 shows the correlation between the NfL and MMSE score and the different categories of the MMSE. There was no correlation between the concentration of NfL and MMSE among total population (*r* = −0.153, *p* = 0.277). Figure 4 illustrated the correlation between the NfL and total MMSE in patients with (*r* = 0.137, *p* = 0.583) or without cognitive impairment (*r* = 0.155, *p* = 0.333).

**TABLE 6:** the correlation between and NfL and MMSE in total population.

	**Coefficient of correlation**	***p*-value**
MMSE	−0.153	0.227
Orientation to time	−0.061	0.631
Orientation to place	−0.262	0.036*
Registration	−0.038	0.763
Attention and calculation	−0.195	0.123
Recall	−0.058	0.647
Language	−0.042	0.739

## Discussion

Our cohort study revealed that the serum concentration of albumin is lower in patients with ESRD with cognitive impairment than in those with ESRD without cognitive impairment. The serum glucose concentration was higher in the patients with ESRD with cognitive impairment than in the controls. Among the serum biomarkers associating with cognitive impairment, the plasma concentrations of tau protein, Aβ1/40, and Aβ1/42 were similar between the groups. The NfL concentration was marginally higher in the patients who had ESRD with cognitive impairment than in the controls. There was no correlation between NfL, biochemical parameters and total MMSE.

### Diagnostic Efficacy of the Scale for Cognitive Impairment in Patients With CKD

The scale for diagnosing cognitive impairment has been mostly discussed regarding its use for patients with CKD/ESRD. Commonly used scales include the MMSE, modified MMSE, MoCA, Mini-Cog, Digits Symbol Substitution, and Trial B. The aforementioned screening tests are used in different stages of CKD. Drew et al. showed that all the aforementioned screening tools have predictive value for cognitive impairment diagnosis in patients with ESRD receiving hemodialysis. MoCA had the best predictive value, with an AUC of 0.81 (95% CI: 0.730–0.890) (Drew et al., 2020). In contrast with other scales, MMSE serves as the screening assessment for patients with cognitive impairment (O’Bryant et al., 2008). In our study, MMSE score ≤ 24 was the definition of cognitive impairment adopted in this study. During the test, the MMSE was applied within the first hour of hemodialysis to avoid the influence of brain perfusion due to ultrafiltration during treatment. Moreover, categories involving memory, execution, and appraisal were lower in the cognitive impairment group than in the control group. Our results demonstrated that the education years less than 6 years were more common in the patients with cognitive impairment, which was coherent with studies on the MMSE and education level (Crum et al., 1993; Matallana et al., 2011). Therefore, other screening or diagnostic scales might be needed along with MMSE in the future studies.

### The Role of NfL for Risk Assessment for Cognitive Impairment

Neurofilament is the cytoskeleton of neuron axons. On the basis of size and caliber, the neurofilament could be categorized as light (6 kDa), medium (145 kDa), or heavy (200 kDa). Studies have shown that myelinated neuron damage is common among patients with ESRD because of altered blood perfusion. Findlay et al. stated that the cerebral mean flow velocity decreases during dialysis in a time-dependent manner, and such a decline reflects the progression of white matter hyperintensity (Findlay et al., 2019). The decrease in cerebral blood flow velocity was correlated with MoCA score in patients on dialysis. Ultrafiltration during renal replacement might influence brain perfusion. Thus, the axons within white matter are vulnerable during ultrafiltration, and axon damage might explain the increase in plasma NfL. Additionally, sympathetic hyperactivity is a common phenomenon in patients with ESRD. In patients with ESRD, inflammation, hyperactivity of the renin–angiotensin–aldosterone system, or insulin resistance causes sympathetic hyperactivation (Kaur et al., 2017). Insufficient parasympathetic tone reduces acetylcholine release within the synaptic cleft. Sato et al. reported that cardiovascular mortality was low when treating patients with amnesia who were administered an acetylcholine esterase inhibitor (Sato et al., 2010). The transmitral flow in diastolic refilling was impaired in patients with dementia who were not treated with acetylcholine esterase inhibitors. Thus, sympathetic hyperactivity might contribute to cognitive impairment (Vistisen et al., 2014; Nicolini et al., 2020). The percentage of patients with diabetes mellitus was higher in the cognitive impairment group than in the control group (78.2% vs. 58.5%). The NfL concentration was high in patients with diabetes mellitus during hypoglycemia and was negatively correlated with the gray matter volume of the frontal lobe (Sampedro et al., 2020). In our study, the NfL concentration was marginally higher in the cognitive impairment group and the AUC for diagnosis was 0.687. The correlation between NfL and other biochemical parameter was unable to be made. The possible explanation might be the multiple comorbidities in ESRD patients, such as unmeasured uremic toxin contributing to neuroinflammation within the glial cells (Adesso et al., 2017) or the hyperglycemic status disturbing morphology of neuronal structure (Flores-Gómez et al., 2019). The higher percentage of diabetes mellitus in cognitive impairment group might influence the performance of NfL.

In our cohort, NfL concentration was only negatively correlated with the category of orientation to place in the MMSE, but not total MMSE. In patients with Alzheimer disease or mild cognitive impairment, degenerative change occurs in the hippocampus and is a pathological hallmark, and its synaptic connection within a specific lobe, such as the prefrontal lobe, could directly influence the excitatory potential of neurons (Dégenètais et al., 2003). Moreover, recent reports have provided similar results. For instance, Dinomais et al. (2016) reported that MMSE score was correlated with gray matter damage within the limbic system. Several studies have investigated whether MMSE scores are correlated with damage within a specific area of the brain. Through the use of a 3D mapping technique, the MMSE was correlated with gray matter integrity in the frontal lobe, temporal lobe, and angular gyri (Apostolova et al., 2006). When different categories of the MMSE are being considered, deficit in a specific category could be correlated with specific cortical loss. Vasquez demonstrated that visual attention deficit was correlated with unilateral partial hypoperfusion (Vasquez et al., 2011). The superior and middle gyri of the frontal lobe are the functional areas during calculation (Wang and Wang, 2001). Moreover, Han et al. (2020) reported that patients with stroke with frontal cortex involvement had a lower MMSE score in the calculation category than healthy controls after adjustment for age. Since the correlation between NfL and the total MMSE was lacking, the causes for the cognitive impairment in ESRD might be more than neurodegeneration.

### Plasma Amyloid Beta or Tau Protein Were Not Associated With Cognitive Impairment in ESRD

Traditionally, Aβ aggregation and the hyperphosphorylation of tau protein are important pathogeneses of Alzheimer disease. In Alzheimer disease, the amyloid precursor protein within the cell membrane is cleaved by β- and γ-secretase, which forms insoluble amyloid monomers (Aβ1/42) as the plaque within the brain parenchyma. The soluble monomer Aβ1/40 reflected the less amyloid plaque formation within brain parenchyma (Chen et al., 2017a). As amyloid plaque deposition increases, the spread of phosphorylated tau within neurons is accelerated, forming a neurofibrillary tangle within the brain (Busche and Hyman, 2020). As tau or amyloid formation increases within the brain, the blood–brain barrier effluxes these pathological proteins into the plasma (Liu et al., 2012). *In vivo* research has suggested that the peripheral clearance of pathologic proteins could improve memory (Jin et al., 2017). The concentrations of tau protein and Aβ1/42 were similar in the cognitive impairment group than in the control group. One possible mechanism is that the efflux ability of the blood–brain barrier might be disturbed by protein-bounded uremic toxins (Bobot et al., 2020), thus interfering with biomarker detection. Second, other contributing factors might potentiate neuronal damage in patients with cognitive impairment, such as hypoalbuminemia and anemia.

### Role of Hypoalbuminemia and Anemia in ESRD Patients With Cognitive Impairment

Hypoalbuminemia and anemia have been found to be contributing factors for Alzheimer disease (Llewellyn et al., 2010; Hong et al., 2013). Hypoalbuminemia is common in patients with ESRD. Malnutrition is common in patients with ESRD due to chronic inflammation, anorexia due to poor oral intake, hypercatabolism, and the gradual loss of amino acids during renal replacement therapy (Tonbul et al., 2006). In our study, the body mass index values were similar between the groups, confirming that the results were not confounded by obesity or underweight. Doorduijn et al. (2019) showed that patients with Alzheimer disease or mild cognitive impairment had a high incidence of malnutrition and less fat-free mass. Furthermore, CSF tau concentration was negatively correlated with fat-free mass and malnutrition severity (Doorduijn et al., 2019). In patients with Alzheimer’s disease, malnutrition was closely associated with hyperhomocysteinemia (Sun et al., 2017). Homocysteine activates the phosphorylation of tau protein through protein phosphatase inactivation (Zhang et al., 2008). Renal anemia is caused by insufficient EPO production by tubulointerstitial cells and the resistance of erythropoietic precursor cells to EPO due to the accumulation of uremic toxins and systemic inflammation (Batchelor et al., 2020). Vinothkumar et al. (2019) reported that the phosphorylated tau protein concentration was lower in patients with CKD treated with EPO than subjects without EPO treatment. Anemia was associated with neuron function in patients with CKD. Our previous study demonstrated that hemoglobin concentration was negatively correlated with striatum function in patients with ESRD (Hou et al., 2020). Mazumder et al. (2019) reported that mitochondrial dysfunction and oxidative stress accumulation within the brain were the main molecular mechanisms involved in cognitive impairment in an adenine-induced CKD animal model. Insufficient oxygen supply due to anemia might contribute to reactive oxygen species generation. EPO could increase tau phosphorylation during exposure to β-amyloid peptides (Sun et al., 2008). Our study showed that hypoalbuminemia and anemia might be associated with cognitive impairment, but further studies linking these risk factors with pathological characteristics, such as neurofibrillary tangle or amyloid plaque, may be required to determine the mechanism of cognitive impairment in patients with ESRD/CKD.

### The Limitation of the Study

In our study, the average age of patients was higher in the ESRD with cognitive impairment group than in the other group. Furthermore, the cognitive impairment group had a higher proportion of women than the other group. Studies have shown that patients undergoing dialysis tend to belong to the elderly population (Yang et al., 2008; Stevens et al., 2010). We did not rank patients according to cognitive impairment severity or adjust for education level because of the low number of cases. Our cohort study used MMSE to group the subjects. However, MMSE alone might not be sufficient to distinguish the mild cognitive impairment or prodromal dementia (McKhann et al., 2011). Other sophisticated neurospsychological measurements might be needed in addition to MMSE. Second, we chose to test for correlations between peripheral neurological biomarkers and traditional risk factors for cognitive impairment. Protein detection in plasma and CSF has been validated in previous studies. Although CSF and plasma measurements have been verified in other cohort studies, the association between the plasma and CSF neurological biomarkers has not been validated for patients with ESRD. Third, to correlate the peripheral biomarkers with radiologic studies was not performed. No image studies such as magnetic resonance image was performed during the study. The radiologic studies might provide further correlation between the pathologic change of the brain parenchyma and MMSE categories. Fourth, we didn’t exclude the possible AD pathologic change by measuring Aβ and tau in the CSF or by positron emission tomography (Okamura et al., 2014). However, from the study by Gronewold et al. (2016), the CKD might contribute to elevated plasma Aβ concentration. It is still unknown if there is interaction between CKD and the pathologic change in AD. Further analysis of the correlations between plasma neurological biomarkers and the results of radiological studies may be warranted in the future. Finally, the depressive mood and hormonal dysregulation (such as hypothyroidism or growth hormone, which could influence cognitive function, were also the common comorbidities in CKD/ESRD patients (Drew et al., 2019; Rhee, 2019). In this cohort study these comorbidities were not excluded. In the future studies, these confounding disorders should be taken into consideration in the studies related for cognitive impairment in ESRD.

## Conclusion

This case–control study compared the biomarkers for ESRD patients with and without cognitive impairment. Plasma NfL was marginally higher in the cognitive impairment group; the concentration of tau protein, Aβ 1/42, Aβ 1/40 were similar between groups. There was no correlation between NfL and biochemical parameters or total MMSE. Further studies, especially radiological studies, may be necessary to verify the role of these neurological biomarkers and the possible pathogenesis of cognitive impairment in patients with ESRD.

## Data Availability Statement

The raw data supporting the conclusions of this article will be made available by the authors, without undue reservation.

## Ethics Statement

The studies involving human participants were reviewed and approved by the Cardinal Tien Hospital. The patients/participants provided their written informed consent to participate in this study.

## Author Contributions

Y-CH drafted the manuscript and obtained informed consent from the participants. C-LH and C-LL executed statistical analysis. C-MZ, Y-FL, and K-CL conducted the study. Y-LC was responsible for sample collection. R-MC designed the study. All authors contributed to the article and approved the submitted version.

## Conflict of Interest

The authors declare that the research was conducted in the absence of any commercial or financial relationships that could be construed as a potential conflict of interest.

## Abbreviations

Aβ: amyloid beta
AUC: area under the curve
CKD: chronic kidney disease
CSF: cerebrospinal fluid
CI: confidence interval
EPO: erythropoietin
ESRD: end-stage renal disease
GFR: glomerular filtration rate
IMR: immunomagnetic reduction
MMSE: Mini-Mental State Exam
MoCA: Montreal Cognitive Assessment
NfL: neurofilament light chain
ROC curve: receiver operating characteristic curve.
